# Irreducible Posterior Shoulder Dislocation With Concomitant Fracture of Both the Greater and Lesser Tuberosity: An Extremely Rare Shoulder Injury

**DOI:** 10.7759/cureus.52312

**Published:** 2024-01-15

**Authors:** Giannis Kotsalis, Aristidis Georgountzos, Ioannis Kechagias, Maria Ladogianni

**Affiliations:** 1 First Orthopaedic Department, Gennimatas General Hospital, Athens, GRC; 2 Fifth Orthopaedic Department, KAT General Hospital, Athens, GRC

**Keywords:** reduction, lesser tuberosity, greater tuberosity, fracture, posterior dislocation, shoulder

## Abstract

Posterior shoulder dislocation is a relatively rare injury representing only 5% of all shoulder dislocations. It is usually the result of a high-energy trauma or an epileptic seizure. Diagnosis is challenging with half of these injuries missed in the emergency room (ER). Often the dislocation is accompanied by a lesser tuberosity fracture as a result of the impact between the posterior glenoid and the proximal humerus. Additionally, fractures of the greater tuberosity or even the metaphysis are extremely rare, and their treatment remains challenging. We present a rare case of posterior locked shoulder dislocation with a concomitant lesser and greater tuberosity fracture in a young patient. A 29-year-old male was brought to the ER following a motor vehicle accident. The patient reported significant pain and inability to move his left shoulder. The arm was locked in an internal rotation and was neurovascularly intact. Simple radiographs revealed a locked posterior dislocation with fractures of both the lesser and greater tuberosity. The CT scan confirmed the fracture pattern and excluded metaphyseal fracture. Surgical treatment was decided. Under general anesthesia and a classic thoracodeltoid approach, both tuberosities were recognized and the dislocation was gently reduced. Fixation of the tuberosities with an anatomic plate and Ethibond No. 5 sutures was performed. He was discharged the next day with a 30-degree abduction sling cast. He was instructed to perform passive shoulder and scapula exercises once a day. After one month, the sling was removed and active elevation and rotation exercises were started. Plane X-rays were performed at one, six, and twelve months. The reduction remained stable and the patient recovered full range of motion with a slight loss of external rotation (10 degrees) compared to the contralateral limb. He returned to his previous activities without any complications. Posterior shoulder dislocations with additional fractures of the tuberosities are rare and severe injuries requiring open surgery as the humeral head may remain locked prohibiting closed reduction. Even after the reduction, the stable fixation of the tuberosities is crucial for shoulder stability and postoperative clinical and functional results.

## Introduction

Posterior shoulder dislocation (PSD) is a rare injury representing only 5% of all shoulder dislocations. The total annual incidence is 0.6/100,000 and is a result of high-energy trauma or a seizure, with forces acting in a flexed adducted and internally rotated arm [1]. In most traumatic cases, a subchondral fracture of the humeral head is present (reverse Hill-Sachs (RHS)) [2]. The proximal humerus comes in contact with the posterior glenoid rim and results in a locked irreducible dislocation.

Most patients present in the emergency room (ER) with pain in the injured shoulder and an inability to externally rotate their arm. Classic anteroposterior (AP) radiographs are often unreliable, and half of the cases are lost in the ER. The lightbulb sign in the AP radiograph and the axillary Velpeau view can set the diagnosis of a posterior dislocation; however, a CT scan is often required to determine the extent of the injury and any additional fractures [3,4].

RHS can be a subchondral fracture or a large lesser tuberosity (LT) fracture and is associated with recurrent posterior instability of the shoulder. Locked dislocations should be reduced under anesthesia, even though closed reduction is often inevitable and an open procedure is required [5]. RHS lesions are treated after the reduction with subscapularis or lesser tuberosity transfer (Mclaughlin procedure) and LT fractures should be reduced and internally fixed. The combination of posterior dislocation with a greater tuberosity (GT) fracture is an extremely rare condition with just a few reported cases [6,7]. We present a case of posterior locked shoulder dislocation with LT and GT fracture in a young male patient. To our knowledge, no such other injury combination or treatment has been reported in the literature.

## Case presentation

**Figure 1:**
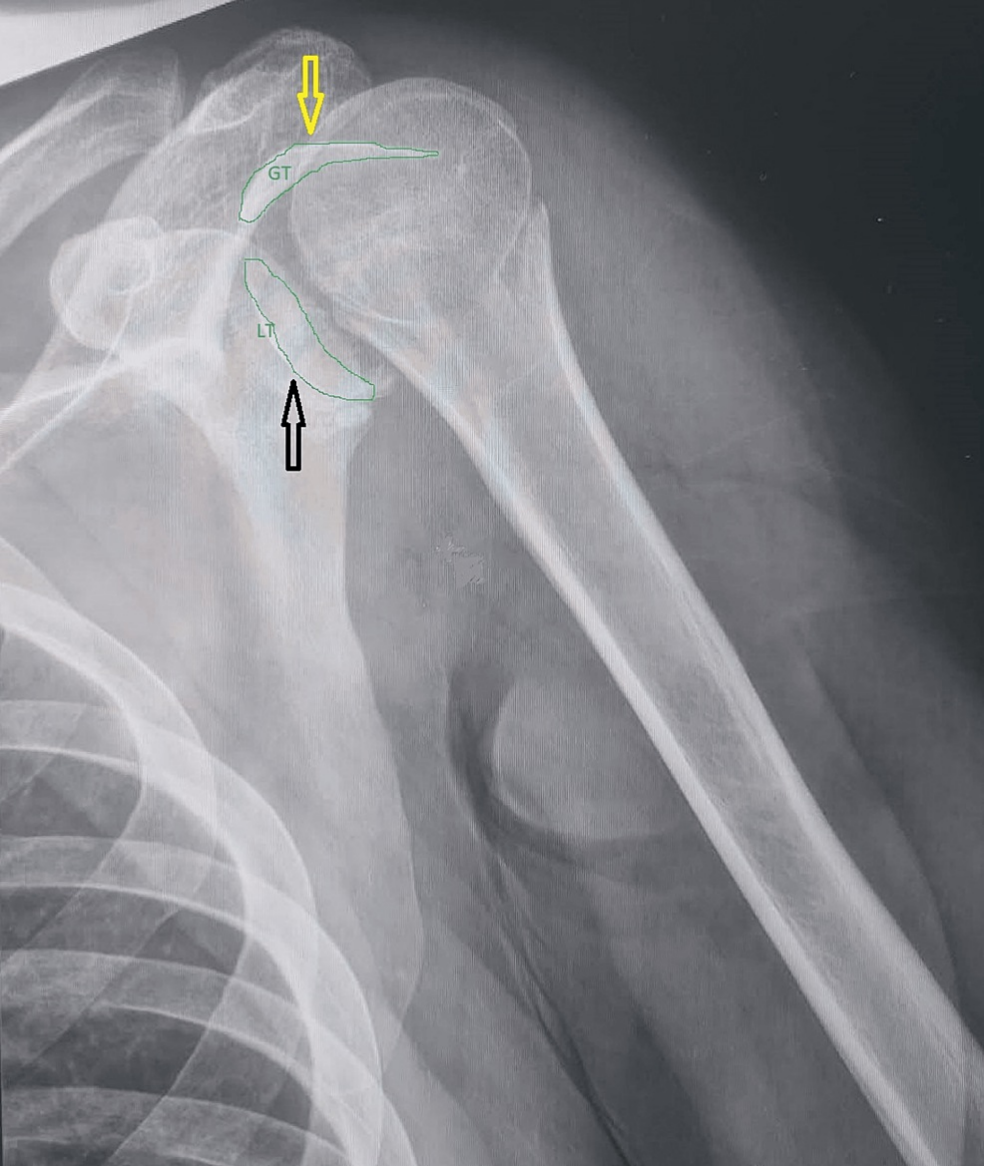
Anteroposterior shoulder X-ray. The greater tuberosity and lesser tuberosity are fractured and displaced. The humeral head is on the same axis as the metaphysis because of the internal rotation (light bulb sign), indicating a possible posterior dislocation. Black arrow: lesser tuberosity. Yellow arrow: greater tuberosity.

A 29-year-old male patient was transferred to the emergency department after a road traffic accident. He suffered a fall from his motorcycle after a collision with a car. He complained of severe pain and functional disability in his left shoulder. On clinical examination, he had pain and obvious deformity of the shoulder, and he was unable to move his arm in any direction, especially in external rotation. He presented intact neurosis with no numbness in the regimental badge region of the axillary nerve while demonstrating normal radial pulse at the wrist. Radiological examination displayed a posterior shoulder dislocation with a fracture in both the LT and GT (Figure 1).

**Figure 2:**
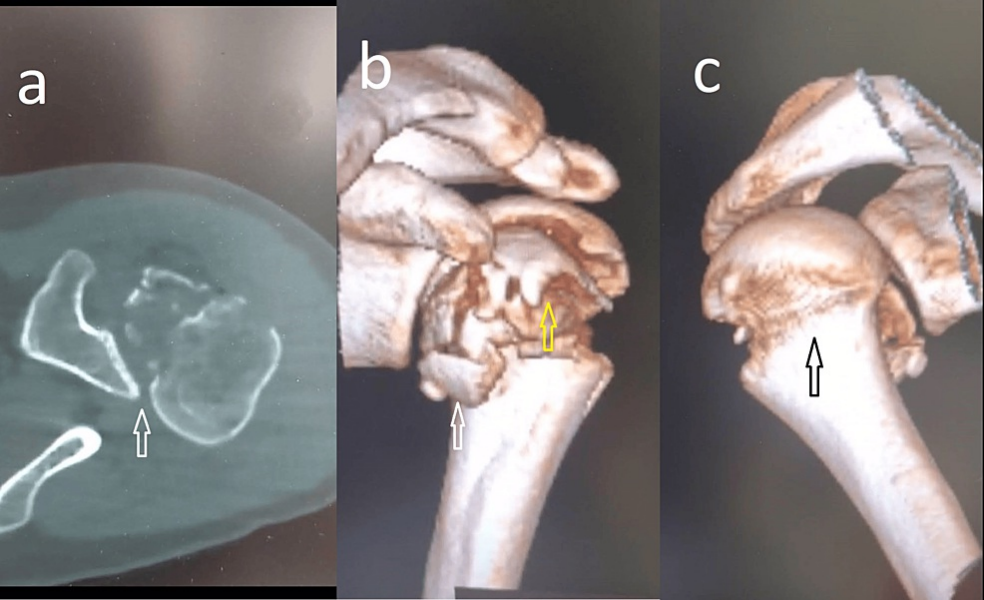
CT scan confirming the posterior dislocation (a), the fracture pattern (b), and the metaphyseal fracture was excluded (c). (a) Posterior dislocated humeral head relative to the posterior glenoid rim (white arrow). (b) Fracture pattern with a fracture of the lesser tuberosity (white arrow) and the greater tuberosity (yellow arrow). (c) Intact metaphysis (black arrow).

He had no other skeletal or visceral injuries. A CT scan was performed where the fracture pattern was confirmed and a metaphyseal fracture was excluded (Figures 2a-2c).

The patient was transferred to the operative theater for closed manipulation under conscious sedation to reduce the dislocation and avoid neurological compromise. Despite prompt sedation and muscle relaxation, the shoulder was still unable to interpose.

**Figure 3:**
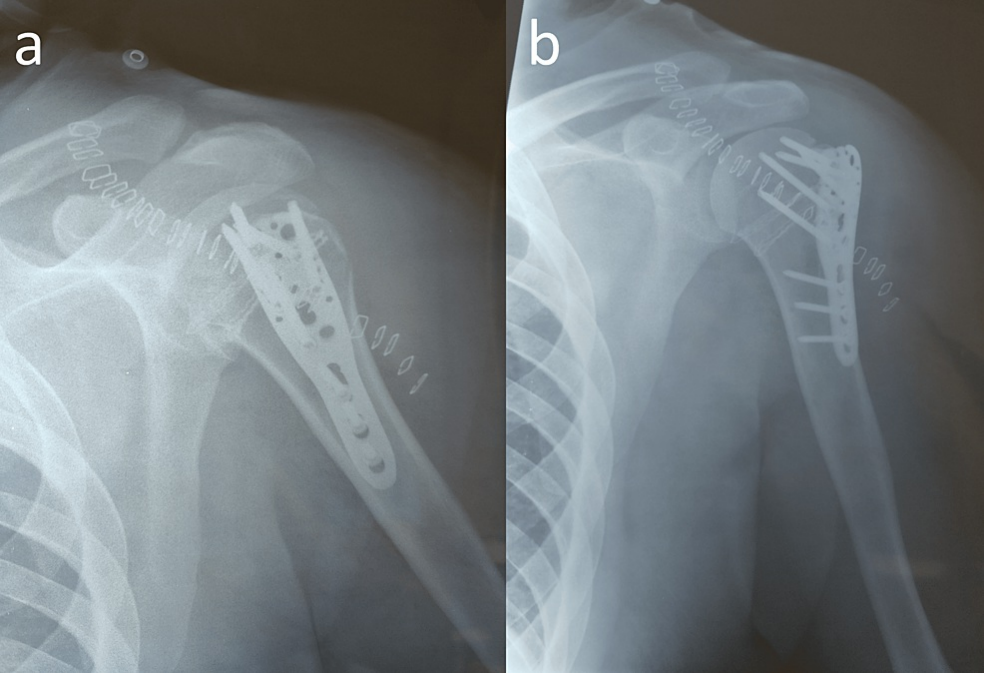
Anteroposterior radiographs of the shoulder with the anatomic plate applied. (a) Radiograph with the arm in internal rotation. The shoulder is reduced and the tuberosities are fixed. (b) Radiograph in external rotation with the arm in external rotation. Both tuberosities are anatomically reduced and fixed.

Open reduction was accomplished thereafter in a beach chair position using the thoracodeltoid approach under general anesthesia. Tenotomy and tenodesis of the biceps tendon were performed using a bone anchor. With the biceps tendon as a guide, the rotator interval was recognized with the two fractured tuberosities on each side. Using two heavy sutures (Ethibond No. 5), both tuberosities were stitched allowing their manipulation during the surgery. By carefully mobilizing the LT along with the subscapularis, an osteotome was positioned between the posterior glenoid and the humeral head. Using the osteotome as a lever to unlock the humeral head, the arm was externally rotated and slightly abducted resulting in humeral head reduction inside the glenoid. Both tuberosities were reduced and an anatomic proximal humerus plate was applied. The plate was preloaded with two pairs of heavy non-absorbable sutures (Ethibond No. 5) and secured in place using three distal and five central locked screws (Figures 3a, 3b).

Following fixation the preloaded sutures were passed through both tuberosities and tied in place. The joint appeared stable in full range of motion.

The patient had an uneventful postoperative recovery without neurovascular compromise. He was discharged the next day with a 30-degree abduction sling cast and the arm in a neutral position. After the first week, passive shoulder and progressive range of motion exercises were started. The patient was instructed to perform passive shoulder and scapula exercises once a day. After one month, the sling was removed and active elevation and rotation exercises were started. Post-reduction radiographs, consisting of AP and lateral views, confirmed that the shoulder was satisfactorily reduced. Repeat X-rays two weeks after the injury confirmed maintenance of reduction. The follow‐up period was 12 months. Radiographs were also obtained at one, six, and twelve months. Furthermore, the patient recovered full range of motion with a slight loss of external rotation (10 degrees) compared to the contralateral limb. He returned to his professional activities (auto mechanic) with no complications reported.

## Discussion

Isolated PSD is a rare entity associated with bone laxity, glenoid retroversion, and hypoplasia [8]. In more than 65% of posterior dislocations, bony and subchondral injuries are identified [2]. The incidence of posterior fracture-dislocations is about 0.9% of all shoulder fractures and dislocations [9]. The fracture of the anteromedial portion of the humeral head (RHS) is present in 29% of the cases, and this incidence increases with age, with an 85% incidence in low-demand individuals. The humeral surgical neck is fractured in 55% of the fracture-dislocations, with LT in 42% and GT in 23% [2].

An isolated AP radiograph is inadequate to confirm a PSD. Hawkins et al. noted that all patients presenting in the ER with the arm in an adducted, flexed, and internally rotated position require an additional axillary view [3]. As patients are often unable to abduct their arm, the Velpeau view is reliable in confirming a posterior dislocation [10]. In the presence or suspicion of additional fracture, a CT scan is required to determine the extent of the bone loss or the fracture location and morphology [11].

PSD should be reduced under general anesthesia [5]. A slight retraction of the arm along with a forward force applied to the humeral head is required. All maneuvers must be done gently and external rotation must be avoided to reduce the danger of iatrogenic fracture in the humeral neck or tuberosities [12]. RHS of less than 20% and an injury less than four weeks old are the main positive factors for a successful close reduction [13]. In cases with a bigger RHS lesion and cases of fracture-dislocations, an open reduction is most likely to be acquired. An open procedure is also necessary in the presence of instability after the closed reduction.

Numerous procedures have been described in the literature to deal with posterior fracture-dislocations. The McLaughlin procedure was initially described to treat humeral head defects between 20% and 40% with subscapularis tenotomy and reattachment inside the injured area [14]. The procedure was modified by several studies [15] to deal with larger and older defects with more than 25% size. Osteotomy of the LT and fixation along with the attached subscapularis seem to provide excellent healing and excellent long-term stability [16]. Iliac autograft has also been used in multiple case series [17] to treat severe bone loss. In cases with bone defects larger than 50%, allograft might be unavoidable [18]. Fresh frozen humeral or femoral heads have been used with good clinical results. Multiple fixation methods have been used to keep the graft in place, including screws and anchors [19]. Autograft and allograft procedures can also be performed in combination with a modified McLaughin procedure [20]. However, when the dislocation is associated with a fracture, such as in our case, the treatment of choice remains unclear.

The most common fracture site associated with posterior dislocations is the humeral neck and the LT. The management of the fracture site after reduction is associated with different choices of treatment in the literature. Conservative treatment after spontaneous fracture reduction has been suggested by Ogawa et al. in a series of four cases [21]. They supported internal fixation in cases of fragment displacement of more than 10 mm. Hayes et al. reported a dislocation with an LT fracture treated with internal fixation. They concluded that posterior dislocation with LT fracture is a combination analogous to the anterior dislocation/GT injury. They highlighted that in cases with spontaneous fragment reduction without displacement, these injuries can be treated conservatively. On the other hand, internal fixation allows an earlier and safer mobilization and rehabilitation [22]. In our case, we considered the LT fracture as an equivalent injury to RHS, and its fixation using sutures was expected to contribute to shoulder stability.

The incidence of the posterior dislocation along with GT fracture is extremely low. Zyffahrizat et al. reported a case of a GT fracture along with a posterior dislocation which was treated with open reduction and internal fixation with two partially threaded screws [6]. Mutahame et al. dealt with a posterior dislocation along with a GT fracture that was treated with closed reduction. No additional intervention was required as the GT was in an anatomical position after the reduction [7]. Roileaut et al. reviewed posterior fracture-dislocations and suggested an algorithm for treating these injuries. They proposed a closed or open reduction of the dislocation and re-evaluation of the fracture pattern. In the presence of fragment displacement, they suggested an internal fixation [2]. The fixation method varies through screws, plate fixation, or even arthroplasty in cases of severe humeral head compromisation.

In the presented case both GT and LT appeared fractured and displaced. To our knowledge, this type of injury has not been reported yet. Closed reduction was not possible even after the patient was under anesthesia in the operating room. After the open reduction and with both tuberosities fractured, we treated the injury as a three-part proximal humerus fracture. As the greater tuberosity was a large fragment, we believe that osteosynthesis provided the most stable result. As already mentioned, the reduction and fixation of the LT with sutures and screws provided additional stabilization against a recurrent posterior dislocation. Early mobilization and rehabilitation offered the patient excellent results in terms of functionality and range of motion.

## Conclusions

PSDs along with tuberosity fractures are a rare entity. Higher suspicion and adequate radiographic examination lead to the right and early diagnosis. All reduction attempts must be done under general anesthesia and with gentle maneuvres of adduction and internal rotation. In cases of closed reduction, tuberosities with small displacement can be treated conservatively. In cases where closed reduction is not possible and an open reduction is performed, the fixation of any additional fragments, displaced or not, seems logical as it will likely allow a safer and earlier rehabilitation.

## References

[1] Kowalsky MS, Levine WN (2008). Traumatic posterior glenohumeral dislocation: classification, pathoanatomy, diagnosis, and treatment. Orthop Clin North Am.

[2] Rouleau DM, Hebert-Davies J (2012). Incidence of associated injury in posterior shoulder dislocation: systematic review of the literature. J Orthop Trauma.

[3] Hawkins RJ, Neer CS 2nd, Pianta RM, Mendoza FX (1987). Locked posterior dislocation of the shoulder. J Bone Joint Surg Am.

[4] Koutserimpas C, Piagkou M, Karaiskos I, Chronopoulos E, Arkoudis NA (2023). Posterior dislocation of the shoulder: the light-bulb sign. Cureus.

[5] Walch G, Boileau P, Martin B, Dejour H (1990). [Unreduced posterior luxations and fractures-luxations of the shoulder. Apropos of 30 cases]. Rev Chir Orthop Reparatrice Appar Mot.

[6] Zufahrizzat S, Nuruddin M, Saifudin O, Rauf A (2020). Acute traumatic posterior shoulder dislocation with greater tuberosity fracture. Orthop J Sports Med.

[7] Mutchamee S, Pongsamakthai W (2018). A case report of posterior shoulder dislocation with greater tuberosity fracture. J Southeast Asian Orthop.

[8] McLaughlin HL (1952). Posterior dislocation of the shoulder. J Bone Joint Surg Am.

[9] Robinson CM, Akhtar A, Mitchell M, Beavis C (2007). Complex posterior fracture-dislocation of the shoulder. Epidemiology, injury patterns, and results of operative treatment. J Bone Joint Surg Am.

[10] Bloom MH, Obata WG (1967). Diagnosis of posterior dislocation of the shoulder with use of Velpeau axillary and angle-up roentgenographic views. J Bone Joint Surg Am.

[11] Wadlington VR, Hendrix RW, Rogers LF (1992). Computed tomography of posterior fracture-dislocations of the shoulder: case reports. J Trauma.

[12] Robinson CM, Seah M, Akhtar MA (2011). The epidemiology, risk of recurrence, and functional outcome after an acute traumatic posterior dislocation of the shoulder. J Bone Joint Surg Am.

[13] Rouleau DM, Hebert-Davies J, Robinson CM (2014). Acute traumatic posterior shoulder dislocation. J Am Acad Orthop Surg.

[14] McLaughlin HL (1963). Locked posterior subluxation of the shoulder: diagnosis and treatment. Surg Clin North Am.

[15] Delcogliano A, Caporaso A, Chiossi S, Menghi A, Cillo M, Delcogliano M (2005). Surgical management of chronic, unreduced posterior dislocation of the shoulder. Knee Surg Sports Traumatol Arthrosc.

[16] Castagna A, Delle Rose G, Borroni M, Markopoulos N, Conti M, Maradei L, Garofalo R (2009). Modified MacLaughlin procedure in the treatment of neglected posterior dislocation of the shoulder. Chir Organi Mov.

[17] Khira YM, Salama AM (2017). Treatment of locked posterior shoulder dislocation with bone defect. Orthopedics.

[18] Gerber C, Catanzaro S, Jundt-Ecker M, Farshad M (2014). Long-term outcome of segmental reconstruction of the humeral head for the treatment of locked posterior dislocation of the shoulder. J Shoulder Elbow Surg.

[19] Gerber C, Lambert SM (1996). Allograft reconstruction of segmental defects of the humeral head for the treatment of chronic locked posterior dislocation of the shoulder. J Bone Joint Surg Am.

[20] Kokkalis ZT, Mavrogenis AF, Ballas EG, Papanastasiou J, Papagelopoulos PJ (2013). Modified McLaughlin technique for neglected locked posterior dislocation of the shoulder. Orthopedics.

[21] Ogawa K, Yoshida A, Inokuchi W (1999). Posterior shoulder dislocation associated with fracture of the humeral anatomic neck: treatment guidelines and long-term outcome. J Trauma.

[22] Hayes PR, Klepps S, Bishop J, Cleeman E, Flatow EL (2003). Posterior shoulder dislocation with lesser tuberosity and scapular spine fractures. J Shoulder Elbow Surg.

